# Zn ion-implanted absorbable Fe for improved cytocompatibility and mitigated neointimal hyperplasia

**DOI:** 10.1093/rb/rbaf112

**Published:** 2025-11-07

**Authors:** Xueying Wang, Hongyi Liu, Mingjiang Sun, Aihua Liu, Yan Li

**Affiliations:** School of Materials Science and Engineering, Beihang University, Beijing 100191, China; Beijing Neurosurgical Institute, Beijing Tiantan Hospital, Capital Medical University, Beijing 100070, China; School of Biomedical Engineering, Capital Medical University, Beijing 100069, China; Beijing Neurosurgical Institute, Beijing Tiantan Hospital, Capital Medical University, Beijing 100070, China; Beijing Neurosurgical Institute, Beijing Tiantan Hospital, Capital Medical University, Beijing 100070, China; School of Materials Science and Engineering, Beihang University, Beijing 100191, China; Hangzhou International Innovation Institute, Beihang University, Hangzhou 311115, China

**Keywords:** Fe-based alloy, zinc, biodegradable, neointimal hyperplasia, ion implantation

## Abstract

Ultrathin strut Fe-based scaffolds have made significant progress in the field of vascular interventional devices, but the issues of slow degradation and insufficient biocompatibility at the cell/material interface remain unresolved. This study prepared a functionalized surface on Fe by Zn ion implantation (Zn/Fe). Zn/Fe preferentially releases the antioxidant element Zn in the early degradation due to Zn–Fe galvanic coupling corrosion. It facilitates the adhesion and proliferation of endothelial cells and mitigates the risk of oxidative stress. The degradation rate of Zn/Fe was about 1.6 times that of Fe after 56 days of implantation in the common carotid artery of rats, with a 36.1% reduction in lumen loss rate, demonstrating favorable *in vivo* biocompatibility. These findings provide valuable insights for the development of novel functionalized Fe-based scaffold medical devices.

## Graphical abstract

**Figure:**
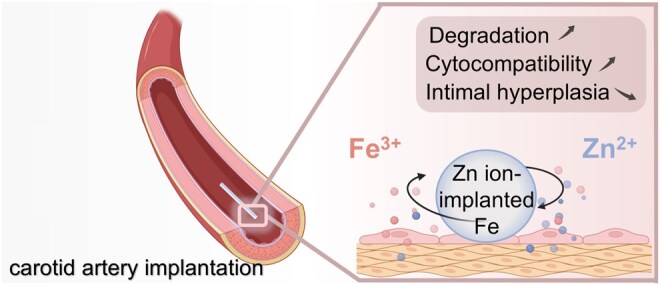

## Introduction

Cardiovascular and cerebrovascular diseases show an increasingly significant impact on human health and the incidence continues to rise. Since the invention of the vascular stent in the 1980s, percutaneous coronary intervention (PCI) has become one of the most effective medical interventions for the treatment of coronary and peripheral artery disease. Fe is regarded as a promising candidate for the preparation of bioresorbable metallic scaffolds due to its excellent mechanical properties, wide body distribution and important physiological functions. Fe-based degradable scaffolds have made significant progress in recent years, demonstrating safety and efficacy in the abdominal aorta and coronary arteries of rabbits and pigs [1–3]. However, the slow corrosion rate of pure Fe limits its application in the field of degradable implantable devices [4–6].

To date, various alloying, severe plastic deformation and surface modification methods have been employed to modulate the degradation behavior of Fe-based alloys [7–10]. The mechanical and corrosion properties were significantly improved by these methods, but some alloy systems showed endothelial activation with inflammation, which could damage the therapeutic efficacy of the implanted devices. Although *in vivo* studies of Fe-based scaffolds have shown no toxic byproducts, the cytocompatibility has been scrutinized due to the generation of strongly oxidizing intermediates such as hydroxyl radicals during the Fenton reaction and their effects on the microenvironment of the biomaterial/cell interface [11, 12]. High levels of reactive oxygen species (ROS) can lead to cellular oxidative stress [13].

Ion implantation technology enables the formation of metallurgical bonding modification layers within the substrate material, exhibiting superior interface stability and deeper affected regions compared to coating modification techniques. This method has been successfully applied to the implantation of various metallic and non-metallic ions, demonstrating significant potential in regulating the corrosion behavior of biomaterials [14–17]. In our recent study, the accelerated corrosion mechanism of Zn ion-implanted absorbable Fe was revealed based on microstructural evolution [18]. The corrosion current density (*i*_corr_) of Zn/Fe was an order of magnitude higher than pure Fe due to the high reaction drive of Zn–Fe galvanic coupling corrosion. The high-energy ion beam generated high-density dislocations and lattice distortions at depths of a few micrometers below the implantation region, rendering Zn/Fe highly corrosion sensitive during the immersion experiment up to 120 days, with an average degradation rate about three times higher than pure Fe. Such a controlled degradation pattern facilitates the early release of the antioxidant element Zn and accelerates degradation following vascular remodeling. The present work focuses on the biological responses of Zn/Fe *in vitro* and *in vivo.* Here, the long-term degradation behavior and neointimal response of Zn/Fe in rat carotid arteries were systematically studied.

## Materials and methods

### Ion implantation process

Flake specimens (10 × 10 × 0.2 mm) with three different implantation conditions were prepared for material characterization and *in vitro* cell studies. Meanwhile, the same implantation procedure was performed for iron wires (Φ 0.23 mm) for *in vivo* implantation. As shown in Table 1, the process parameters used for the ion implantation are based on previous work. Samples modified with Zn ions were designated as Zn/Fe. Due to differences in implantation energy and dose, Zn/Fe1045, Zn/Fe1060, and Zn/Fe2045 exhibited different corrosion rates *in vitro* [18].

**Table 1.** Ion implantation parameters

	Fe	Zn/Fe1045	Zn/Fe1060	Zn/Fe2045
		(General)	(High energy)	(High dose)
Dose (×10^16^ ions·cm^−2^)	/	10	10	20
Energy (kV)	/	45	60	45

### Microstructure characterization

The surface morphology and composition of the implanted wires were characterized by scanning electron microscopy (SEM) and energy dispersive X-ray spectroscopy (EDS).

### Extract preparation

*In vitro* cell experiments were performed to compare the release of Zn and Fe ions in the culture medium under different implantation conditions. Extracts were prepared by immersing samples in a complete medium (90% DMEM + 10% FBS) at a ratio of 1.25 ml/cm^2^ and fully immersed for 48 h in a constant temperature shaker (37°C, 90 rpm). After centrifugation (1000 rpm, 5 min), the supernatant was taken for cell culture and the remaining extract was digested and tested for ion concentration by an inductively coupled plasma optical emission spectrometer (ICP). The high-concentration ionic stock solution was made with FeCl_3_, FeCl_2_, and ZnCl_2_ dissolved in culture medium. Dilute the stock solution with complete medium (CM) before use. The cytotoxicity of the ionic solution was tested with the CCK8 method after 24 h of co-culture with cells.

### Cell study

Human umbilical vein endothelial fusion cells (EA. hy926, ATCC CRL-2922, USA) were used to evaluate the impact of materials on cell adhesion and cytotoxicity. Cells were cultured in Dulbecco’s Modified Eagle Medium (DMEM, Gibco, USA) containing 10% fetal bovine serum (FBS, Gibco, USA). Samples were sonicated with acetone and anhydrous ethanol for 10 min and sterilized under ultraviolet light irradiation in an ultra-clean table for 30 min. Cells were inoculated in 96-well plates at 1 × 10^4^ cells/well and incubated for 24 h at 37°C in a 5% CO_2_ cell culture incubator to allow attachment. The original medium was replaced by extracts. After 24 h of incubation, 100 μL of DMEM medium containing 10% CCK8 (CCK-8, Dojindo, Japan) was added to each well and incubated for 1 h in the cell culture incubator. Absorbance was tested at 450 nm using an microplate reader (Biotek Epoch Elx808, USA). At the same time, sterilized samples were immersed in Hank’s containing 10 mM disodium terephthalate (TA, Aladdin, China) and incubated in a shaker (37°C, 90 rpm) for 48 h following the same procedure as above. The extracts were filtered through a 0.22 μm pinhole filter and fluorescence at 425 nm was measured by a fluorescence spectrophotometer (QM40, PTI).

For cell morphology observation, materials were washed and sterilized as described above and placed in 24-well plates. EA.hy926 cells were inoculated at 5 × 10^4^ cells/well in plates directly co-cultured with the materials. After 6 h of incubation, the surface of the specimen was washed with phosphate-buffered saline (PBS) and fixed in 4% paraformaldehyde for 1 h. Subsequently, the samples were permeabilized with 0.5% Triton X-100 solution for 5 min, followed by 1% BSA blocking for 30 min. FITC (30 min) and DAPI (5 min) staining were performed sequentially. After the staining, cells were rinsed thoroughly with PBS to remove residual dye and photographed with an inverted fluorescence microscope (Olympus, Japan). Immunofluorescence areas of nuclei and cytoskeleton were calculated using ImageJ software. Thresholding was performed using the Default method. All images were measured for fluorescence area under the same threshold and algorithm conditions. The nuclear-to-cytoplasmic (N/C) ratio was defined as the ratio of the area of the nucleus to the cytoskeleton.

### Surgical procedure

Animal tests were performed with Fe and Zn/Fe1060 (Φ 0.23 × 10 mm) wire implants. Zn/Fe1060 wires (*n* = 16) and pure Fe wires (*n* = 12) were sterilized by UV irradiation and implanted into the common carotid artery (CCA) of rats (*n* = 28). All animal studies were approved by the Institutional Animal Care and Use Committee of Beijing Tiantan Hospital (Ethics number: 202203005) and carried out according to international and national law and policies (ARRIVE guidelines and the Basel Declaration, including the 3Rs concept). The SPF (Specific Pathogen-Free) grade adult male Sprague-Dawley rats, weighing 280–320 g, from Vital River (USA) were used for the experiments. All animals were maintained in a humidity (60 ± 5%) and temperature (25 ± 1°C) controlled environment with free access to food and water during a 12-h light–dark cycle. Rats were anesthetized by intraperitoneal injection of pentobarbital (340 mg/kg). The skin of the neck was cleaned and shaved (Supplementary Figure S1a). The skin and muscle tissue were incised with dissecting scissors across the midline (Supplementary Figure S1b). Vascular clips were placed proximally and distally (near the bifurcation of the common carotid artery), at which point blood flow was briefly interrupted. A 1 ml hypodermic needle was used to perforate the vessel close to the vascular clamp under a microscope. After successful perforation, the Fe wire was passed along the orifice into the vessel (Supplementary Figure S1d). The ends of the wire were secured with 12-0 microtape suture needles, and the vessel was repaired by suturing the orifice. Both ends of the hemostat were opened to restore normal blood flow (Supplementary Figure S1e). The skin tissue was sutured (Supplementary Figure S1f). After surgery, the animals were transferred to a heated cage at a temperature of 37.5°C for recovery. Respiration, heart rate, and skin color were monitored every 15 min until the animals recovered to normal, and normal left eye activity was used as an indicator of short-term recurrence after surgery. X-rays were performed 56 days after implantation. All animals survived the implantation procedure.

### Histological analysis

Experimental rats were euthanized and autopsied at the indicated time points. Tissues with implanted wires were fixed in 4% paraformaldehyde and embedded in methyl methacrylate. At least three sections of each sample were selected for observation from the proximal, terminal, and distal ends, respectively. Each slice was cut and ground to a thickness of 6–7 μm for Hematoxylin and Eosin (H&E) staining to assess inflammatory cells and tissue responses. Samples were visualized under a high-quality microscope. Neointimal thickness, neointimal area, and lumen loss rate were quantified using ImageJ software.

### Immunofluorescence staining

Carotid tissue after 10 months of implantation was divided longitudinally into two parts. One part was used for hard tissue sectioning and routine HE staining. The other part was used to remove the implanted wires with forceps under a dissecting microscope. Subsequently, frozen sections and immunofluorescence staining were performed sequentially. The sections were imaged using a microscope slide scanner and analyzed by ImageJ software. The remaining resin-embedded blocks were mechanically ground, polished, and gold-sprayed for SEM characterization and EDS analysis.

### Statistical analysis

The experimental data were analyzed by SPSS 29.0. The data were presented as mean ± standard deviation (*n *≥ 3, independent samples). Student’s independent t-test was used for differences between the two groups. One-way analysis of variance (ANOVA) was used for comparisons of more than two groups, followed by Tukey’s multiple comparisons. The significance of differences was analyzed at levels of *P < 0.05, **P < 0.01, ***P < 0.005 and ****P < 0.001.

## Results

### Adhesion and proliferation of ECs

**Figure 1:**
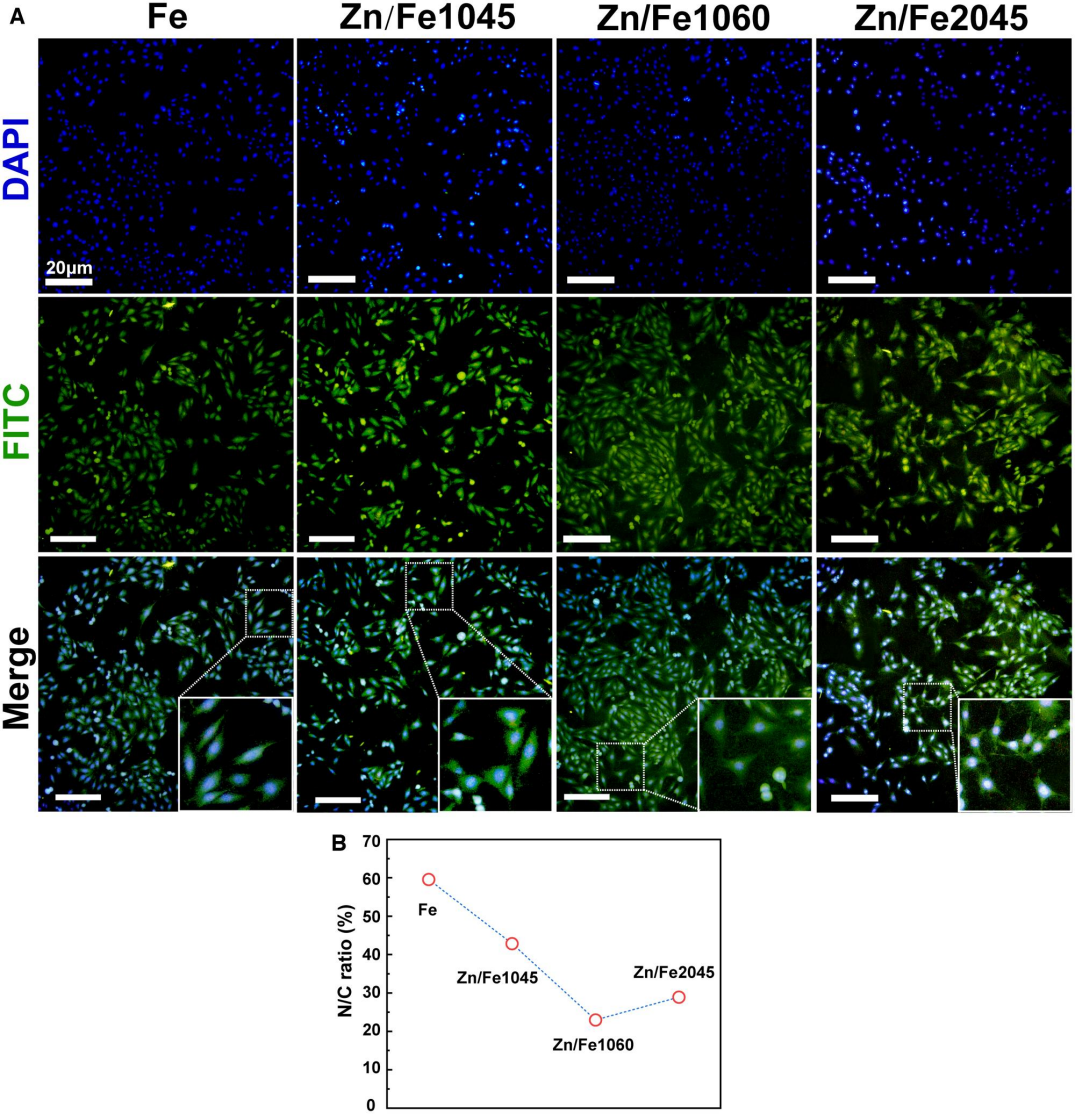
Cell adhesion. (**A**) Immunofluorescence staining images of Fe and Zn/Fe co-cultured with ECs after 6 h. Scale bar is 20 μm; (**B**) Semi-quantitative calculation of nuclear-to-cytoplasmic (N/C) ratio of ECs.

Figure 1 is the immunofluorescence image of endothelial cells (ECs) co-cultured with materials for 6 h. ECs were seeded and adhered normally on the Fe and Zn/Fe surfaces, and no statistically significant difference was observed in the number of adherent cells in various regions. The edge of cells was blurred, caused by corrosion products adhering to the surface of the specimen and affecting the staining. Additionally, ECs on Zn/Fe series samples exhibited a spreading morphology with a more stretched cytoskeleton compared to Fe (Figure 1A). The quantitative results of the nuclear to cytoplasmic (N/C) ratio are shown in Figure 1B, which support the above result.

**Figure 2:**
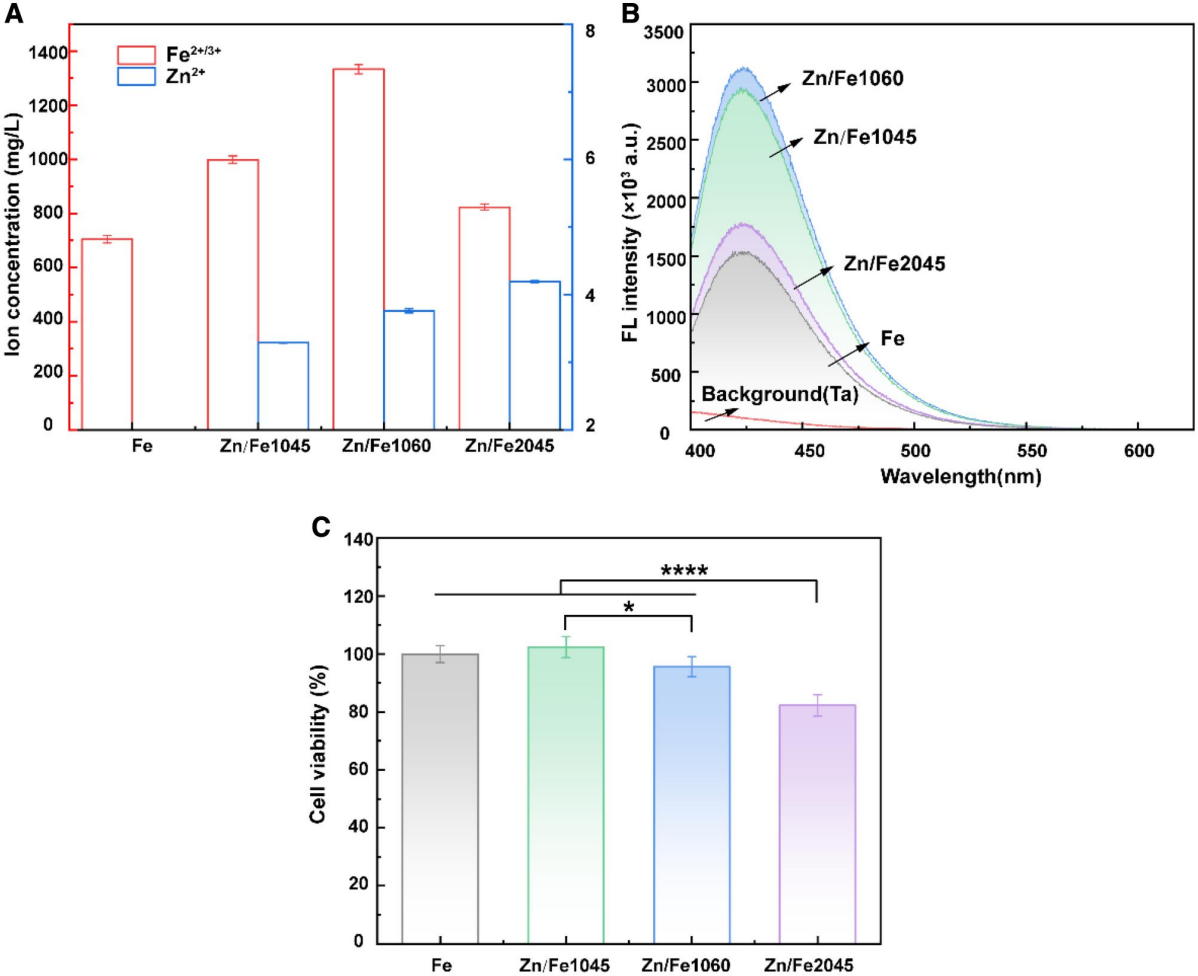
*In vitro* cytocompatibility. (**A**) Ion concentration of the extracts (mean ± SD); (**B**) terephthalate (TA) fluorescence assay; and (**C**) cytotoxicity of Fe and Zn/Fe extracts cultured with ECs for 24 h (*n* = 6, *P < 0.05, **P < 0.01, ***P < 0.005 and ****P < 0.001).

Figure 2 exhibits the composition of Zn/Fe extracts and their effect on the viability of ECs. As shown in Figure 2A, the concentration of Fe ions in Zn/Fe extracts was much higher than that in the Fe extract. Based on Fe ions release, the corrosion rate followed the order Zn/Fe1060 > Zn/Fe1045 > Zn/Fe2045 > Fe, which is consistent with previous findings [18]. It should be noted that the above extract was a mixture containing both Fe^2+^ and Fe^3+^. Fe^2+^ is in a reactive state, which is eventually oxidized to stable Fe^3+^, accompanied by the generation of hydroxyl radicals (·OH). ·OH is an active and toxic reactive oxygen species (ROS). The ·OH in extracts was quantified by terephthalate (TA) fluorescence assay in Figure 2B. 2-Hydroxyterephthalic acid (HTA) produced by the reaction of disodium terephthalate with ·OH, reflects the ·OH concentration. The ·OH-related fluorescence intensity increased with the degradation rate. The ·OH density exhibited a trend consistent with ions release, indicating a positive correlation between accumulated ROS concentration and degradation rate. After co-culturing ECs with the extracts for 24 h, there was no significant difference in cell viability with the Fe group except for Zn/Fe2045 (Figure 2C). Among the three modified samples, Zn/Fe1060 showed the highest cell viability. Due to the higher release of Zn ions, Zn/Fe2045 exhibited the worst cytocompatibility. These results indicate that Zn ion implantation not only accelerated the ion release but also favored the adhesion and proliferation of ECs.

### Antioxidant properties of Zn

**Figure 3:**
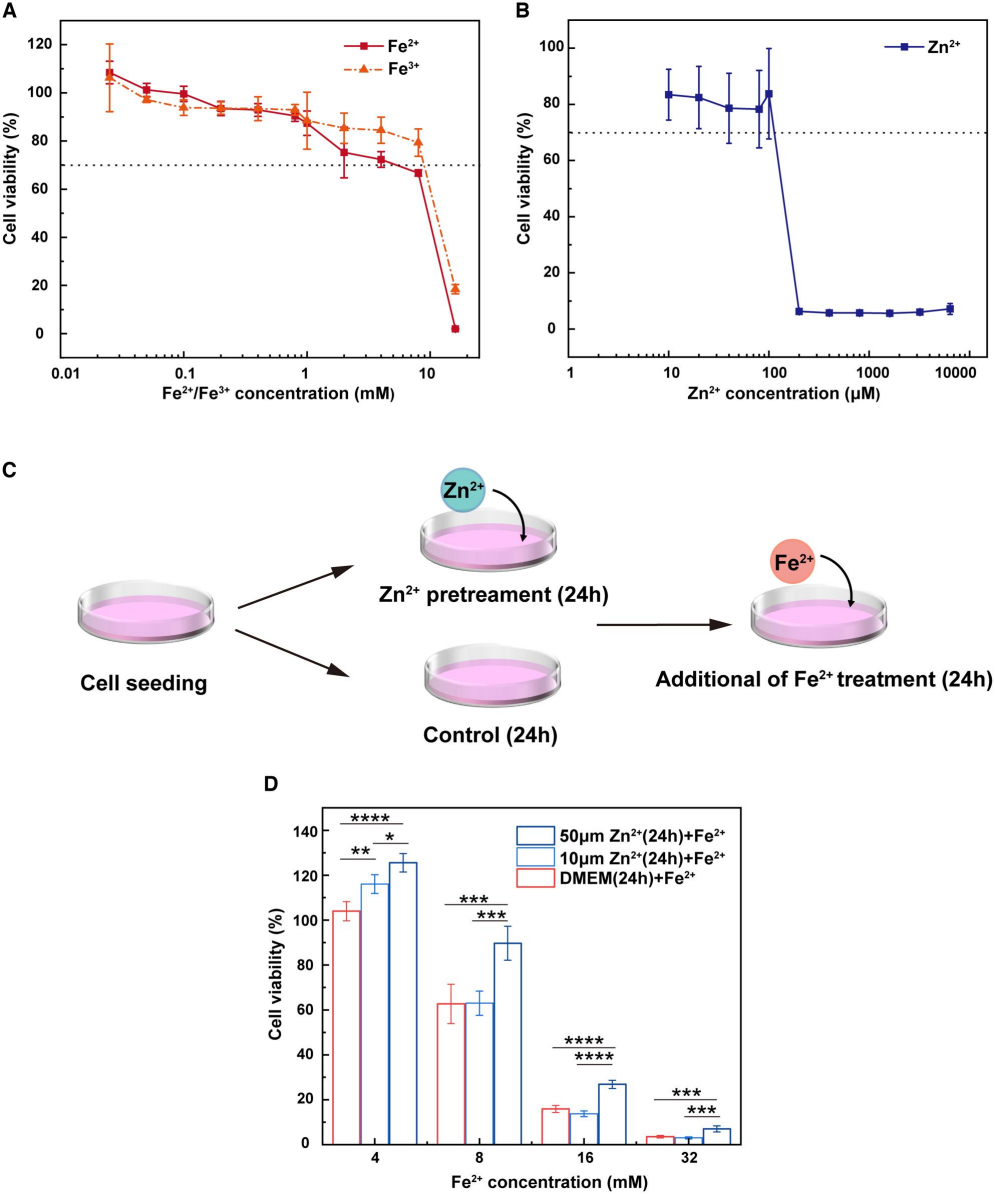
Cell viability exposed to CM containing varying concentrations of (**A**) Fe^3+^, Fe^2+^ and (**B**) Zn^2+^ for 24 h; (**C**) the experimental design scheme; and (**D**) Fe^2+^ cytotoxicity and the effect of Zn^2+^ pretreatment on Fe^2+^ cytotoxicity to ECs determined by CCK8 assay (*n* = 6, *P < 0.05, **P < 0.01, ***P < 0.005 and ****P < 0.001).

It is known that Zn plays an important role in antioxidant defense, which could act as an antioxidant during Fe degradation in various ways to alleviate ROS accumulation in human umbilical vein endothelial cells (HUVECs) [19, 20]. To investigate whether the improved cytocompatibility was related to the antioxidant effect of Zn in the Fe-rich microenvironment, ECs were incubated with ionic solutions to identify the appropriate concentration range. As shown in Figure 3A, ECs viability exhibited a dose-dependent trend in response to Fe^2+^ and Fe^3+^. Significant cytotoxicity (less than 70% relative viability) was exhibited when Fe^2+^/Fe^3+^ concentration reached 8 mM. Moreover, Fe^2+^ showed a stronger inhibitory effect on cell viability at high concentration ranges (2–16 mM) due to its higher chemical reactivity and tendency to oxidize to Fe^3+^, accompanied by ROS generation. Figure 3B shows that the tolerance of ECs to Zn^2+^ was two orders of magnitude lower. No significant effect on ECs viability was observed at Zn^2+^ concentrations below 100 μM. Based on these results, the experimental scheme illustrated in Figure 3C was designed to verify the oxidation resistance of Zn in a Fe-rich microenvironment. As shown in Figure 3D, pretreatment with 10 μM or 50 μM Zn^2+^ significantly increased the ECs viability in the presence of4 mM Fe^2+^ on ECs compared with cells exposed to Fe^2+^ alone. Pretreatment with 50 μM Zn^2+^ significantly reduced Fe^2+^-induced cytotoxicity at concentrations of 8–32 mM. This indicates that Zn^2+^ pretreatment reduced the sensitivity of ECs to Fe^2+^ toxicity-mediated oxidative stress.

### *In vivo* degradation

**Figure 4:**
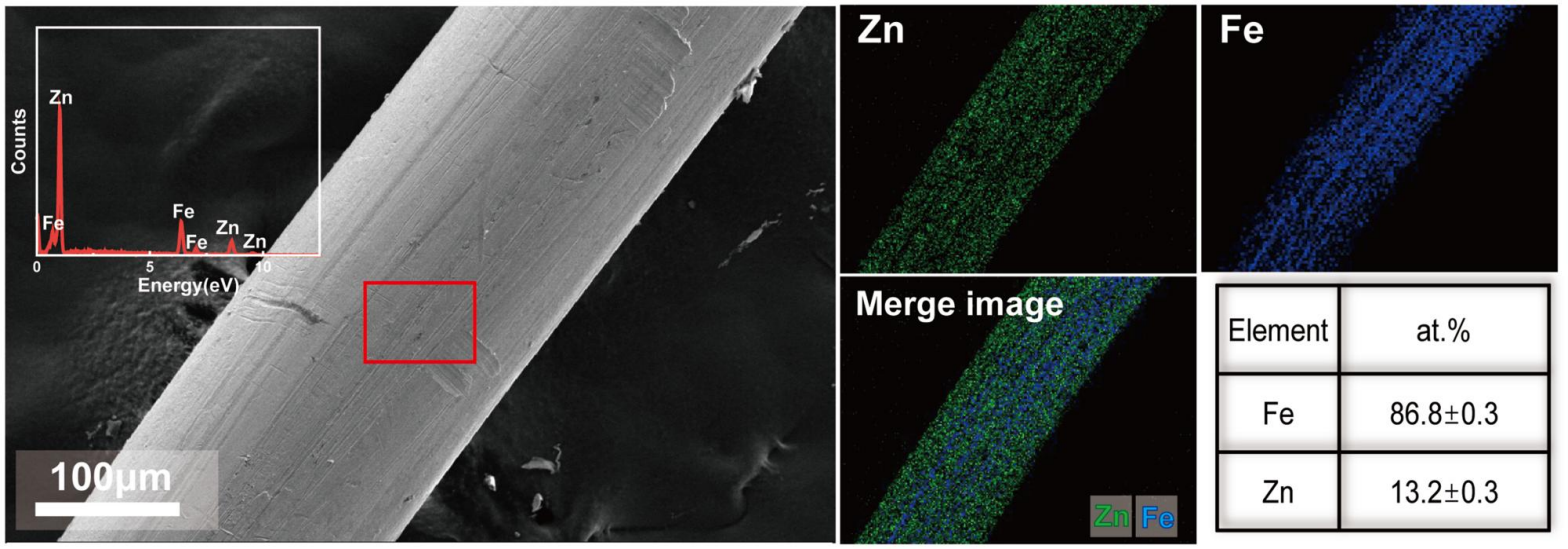
Representative SEM images of Zn ion-implanted Fe wires.

Figure 4 shows the SEM characterization of the pure Fe wire after Zn ion implantation. Elemental Zn (green) and Fe (blue) are uniformly distributed, and the surface concentration of Zn was about 13.2 at.%.

**Figure 5:**
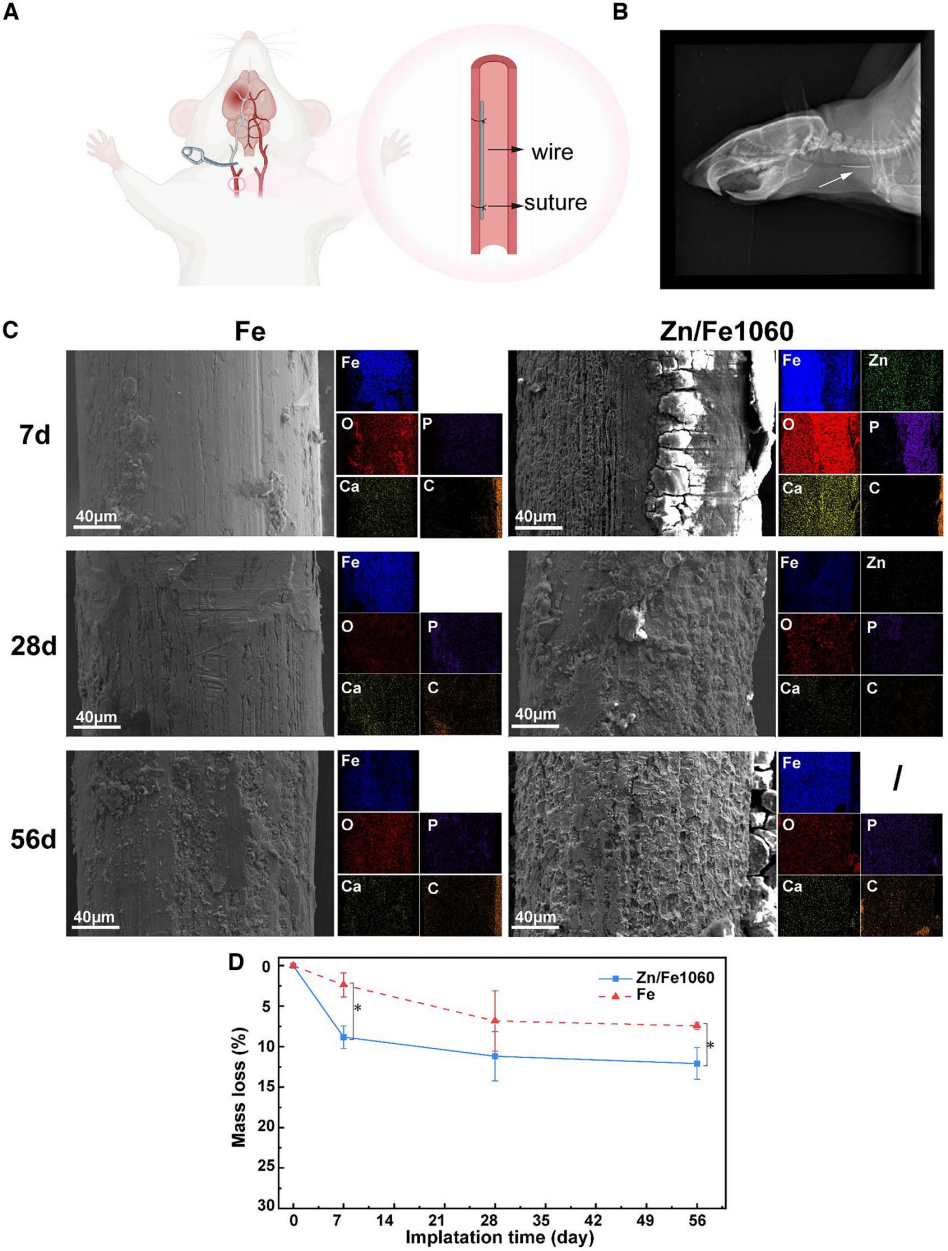
*In vivo* degradation assessment. (**A**) Schematic illustration for the wire deployment in rat common carotid artery (CCA), created in BioRender; (**B**) the representative X-ray image of the rat neck after 56 d of implantation; (**C**) representative SEM images of the Fe and Zn/Fe1060 wires after 7 d, 28 d and 56 d of implantation; and (**D**) mass loss *in vivo* (*n* = 3).

Figure 5 presents the corrosion morphology and degradation behavior of Fe and Zn/Fe1060 wires implanted *in vivo* for 7 d, 28 d, and 56 d. Considering the *in vitro* corrosion performance and cytocompatibility, Zn/Fe1060 was selected for animal experiments with Fe as the control. The rat common carotid artery (CCA) model was utilized to validate the *in vivo* histological response as shown in Figure 5A. Vascular patency was maintained after surgery in all test animals. The 10 mm wire implant in the rat CCA was visible under X-ray (white arrow in Figure 5B). Figure 5C shows the surface corrosion morphology of wires after implantation. A layer of corrosion products was formed on the surface of Zn/Fe1060 after 7 days of implantation, with large-sized longitudinal cracks on it, indicating looseness and easy detachment. EDS showed strong Ga and P signals of the corrosion layer. Localized corrosion pits expanded and interconnected, leading to large-scale detachment of the surface layer at 28 days. The disappearance of the Zn signal in EDS spectra suggested that the surface-modified layer had completely corroded away. After 56 days of implantation, Zn/Fe1060 surface became notably rougher, exposing the fresh Fe matrix beneath the corroded layer. The *in vivo* weight loss curves for Zn/Fe1060 and Fe are shown in Figure 5D. The rate of degradation gradually decreased with time in both groups. At 56 days, the mass loss of Zn/Fe1060 wires was about 1.6 times that of Fe wires (12.1 ± 2.0 wt.% vs. 7.4 ± 0.4 wt.%).

**Figure 6:**
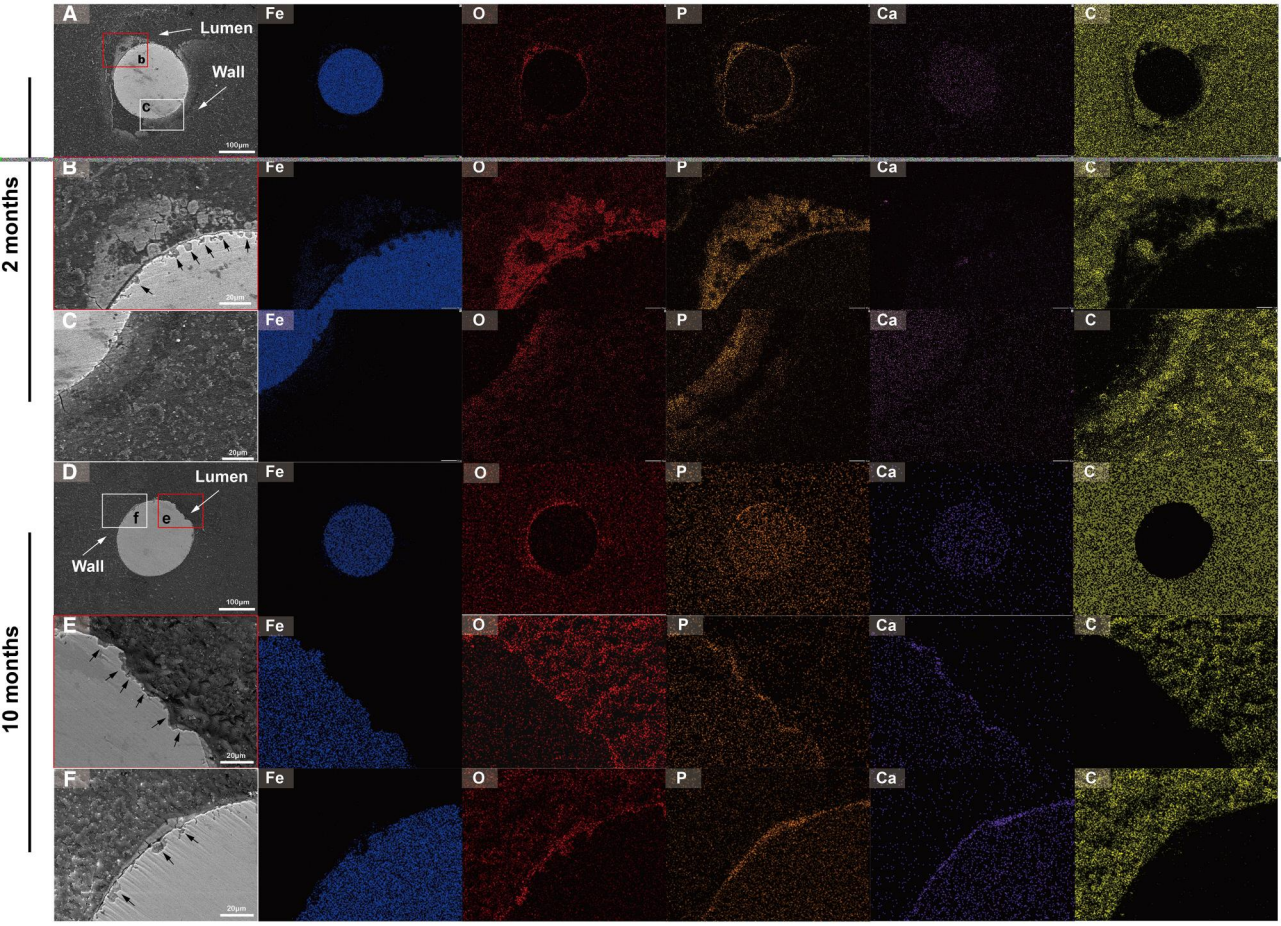
Representative SEM and corresponding EDS images of degraded Zn/Fe wires in rat common carotid arteries after 2 and 10 months of placement.

Figure 6 presents representative SEM images and corresponding EDS elemental mappings of Zn/Fe wire cross-sections implanted for 2 and 10 months. EDS analysis detected signals of Fe, O, C, P and Ca, where O, P and Ca were primarily distributed within the corrosion product layer, and C, P and Ca were also localized in the surrounding vascular tissue. At both time points, the Zn/Fe wires exhibited a homogeneous degradation pattern, but regions close to the lumen and arterial wall exhibited localized corrosion differences. As shown in Figure 6B and E, the regions close to the arterial lumen experienced pronounced corrosion, characterized by a loose corrosion product layer and large corrosion pits (indicated by black arrows). The regions close to the arterial walls were covered by a continuous layer of corrosion products (Figure 6C and F), displaying a lower degree of degradation. Similar to *in vivo* degradation studies for other Fe-based implants, the corrosion products of Zn/Fe wires were mainly composed of Fe and O, derived from the oxide and hydroxide forms of Fe. A distinct Ca/P passivation layer had formed around the Zn/Fe1060 wires after 10 months of implantation (Ca signals in Figure 6E and F).

### Neointimal response

**Figure 7:**
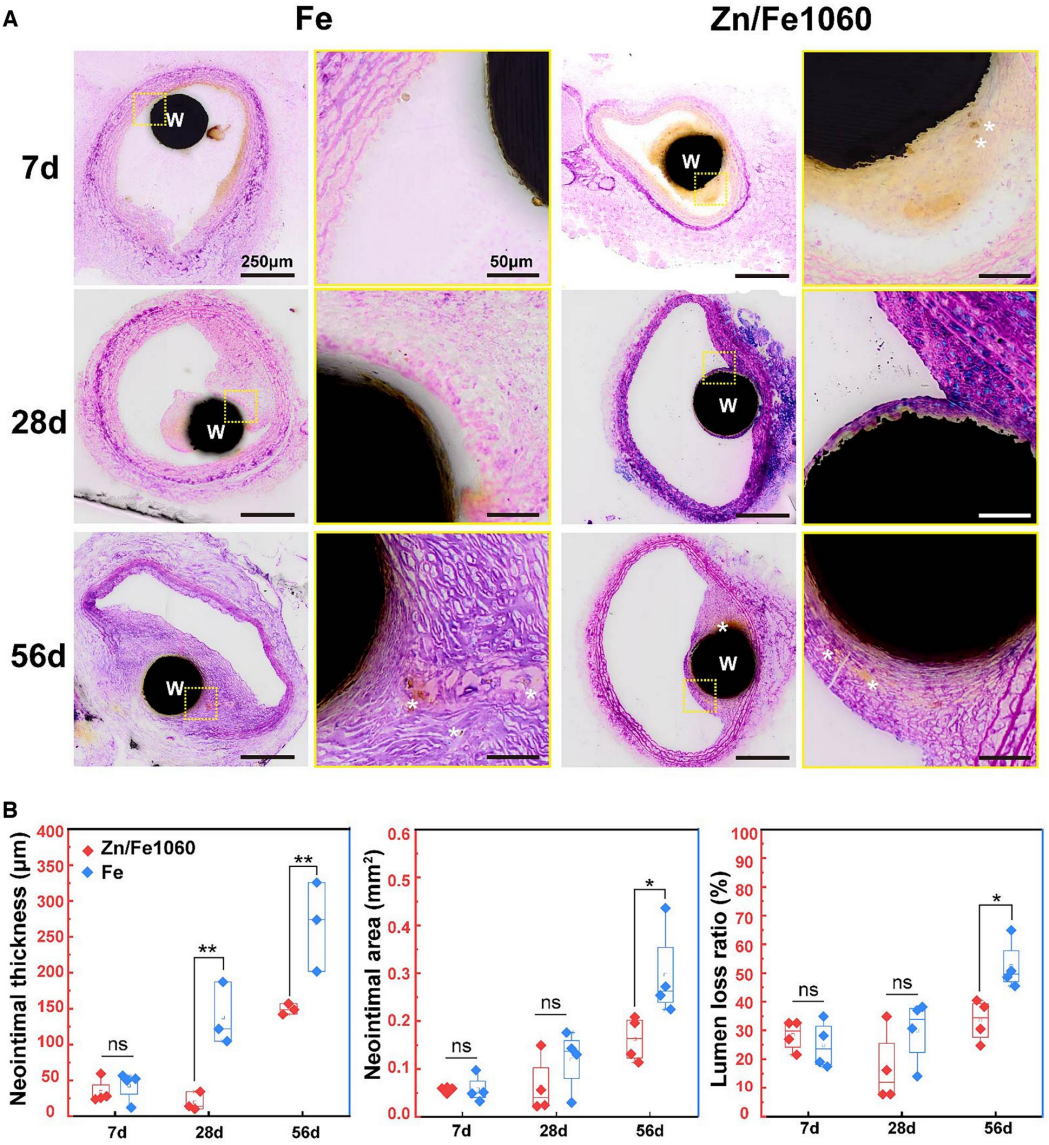
Histologic evaluation. (**A**) Representative H&E staining images of wires implanted rat CCA for 7 d, 28 d and 56 d. (‘W’ indicates the position of the implanted wires. Rectangles indicate magnifications. Scale bars are 250 μm for low magnification (6×) images and 50 μm for high magnification (30×) images). (**B**) Quantitative characterization of the neointimal morphometrics. For box-and-whisker plots, the box edge corresponds to the 25th and 75th percentiles, the inner box line to the 50th percentile, and the whisker line to the maximum-minimum value (the *n* values are shown in the figures, all for independent samples).

Representative H&E-stained cross-section images of wires implanted in rat CCA are shown in Figure 7. Different degrees of brownish-yellow corrosion products accumulated around the implant wires at each time point. Sparse fibrous tissue was observed around Zn/Fe1060 after 7 days of implantation compared to Fe wires. At 28 days, a thin layer of neointima had formed tightly over the Zn/Fe1060 surface, accompanied by mild local inflammation. In contrast, Fe was not fully covered by neointima, and some acellular regions were present demonstrating a heterogeneous reendothelialization process. At 56 days, the neointima was thickened considerably and the alignment of smooth muscle cells was disorganized. No obvious inflammatory reaction was detected and the macrophages containing corrosion products were observed in the neointima (indicated by white asterisks). Figure 7B presents quantitative morphometric analysis of the neointima, including neointimal thickness, neointimal area, and lumen loss ratio. The neointimal thickness of Zn/Fe1060 was significantly lower than that of Fe at both 28 days (19.5 ± 10.6 μm vs. 137.8 ± 35.5 μm) and 56 days (149.1 ± 6.1 μm vs. 267.1 ± 50.8 μm), despite the relatively large standard deviation arising from interindividual variability among animals. The neointimal area (0.16 ± 0.04 mm^2^ vs. 0.30 ± 0.08 mm^2^) and lumen loss ratio (33.5 ± 6.3% vs. 52.4 ± 7.4%) were significantly reduced in Zn/Fe1060 compared to Fe after 56 days of implantation. These results demonstrate that Zn/Fe effectively inhibited neointimal hyperplasia.

**Figure 8:**
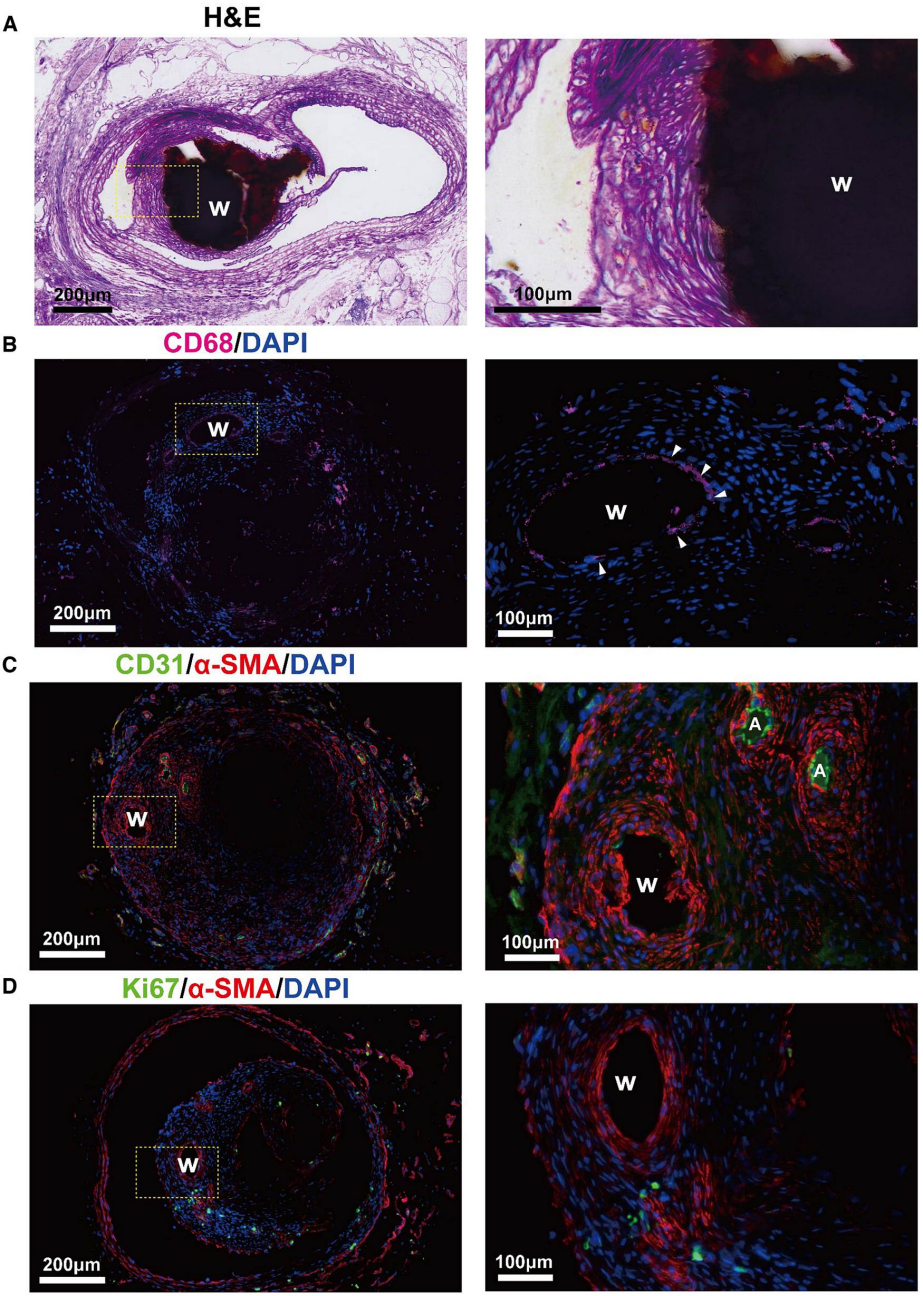
Representative images of (**A**) H&E staining and (**B–D**) immunofluorescence (IFS) staining of vascular cross-sections of rat carotid arteries after 10 months of implantation. (B) IFS staining of macrophage marker CD68 (pink). (C) IFS staining of endothelial marker CD31 (green) and contractile smooth muscle marker α-SMA (red). (D) IFS staining of contractile phenotypic smooth muscle cell marker α-SMA (red) and active proliferating cell marker Ki67 (green). W = wires; A = new vessel in neointima; the right column shows the magnified images of the yellow rectangular box in the corresponding left column of images.

Inflammatory response, excessive smooth muscle cell (SMC) proliferation, and delayed endothelialization are key contributors to neointimal hyperplasia. As illustrated in Figure 8, H&E and immunofluorescence staining (IFS) were performed to assess the inflammatory response, endothelialization and intimal hyperplasia. Figure 8A indicates that the neointima was relatively mature and dense with densely arranged SMCs. The neointima surrounding the Zn/Fe1060 wire consisted of an onion-like luminal layer composed of smooth muscle cells, and a confluent endothelial cells layer. Figure 8B shows macrophages marked by CD68 localized around the corroded wire (white arrows), representing a mild inflammatory response around the implant. CD31 and α-SMA are typical indicators of ECs and contractile phenotype SMCs, respectively. Figure 8C shows that SMCs were well aligned within the neointima and new vessels were formed within the arterial wall, with the outermost layer consisting of a layer of mature ECs. Ki67 markers were used to assess the proliferative activity of neointima. As shown in Figure 8D, most SMCs maintain a contractile phenotype with few Ki67-positive cells detected. These results indicate that Zn/Fe maintained normal vascular function and effectively inhibited neointimal hyperplasia during long-term *in vivo* implantation.

## Discussion

The microstructure and corrosion mechanism of Zn ion-implanted Fe (Zn/Fe) have been reported in our recent work [18, 21]. The mechanism of accelerated corrosion was attributed to the formation of Zn-Fe galvanic coupling and the introduction of high-density defect structures by ion implantation. The objective of this study was to evaluate the *in vitro* and *in vivo* biocompatibility of Zn/Fe and to investigate the biological responses for local cells and tissues.

### Degradation behavior of Zn ion-implanted Fe

Zn/Fe undergoes the following electrochemical reactions during degradation (reactions 1–3).

Anodic reaction:


(1) Zn→Zn2++2e-

(2) Fe→Fe2++2e-

Cathodic reaction:


(3) O2+2H2O+4e-→4OH-

In the initial stage of degradation, the Zn–Fe galvanic coupling corrosion mode is expected to preferentially release Zn^2+^, altering the vascular microenvironment. Zn plays an important role in various biological functions, but the toxicity of Zn^2+^ is dose-dependent [22, 23]. Excessive Zn accumulation can lead to intracellular zinc overload, thereby inducing oxidative stress and resulting in cytotoxic effects [24]. In our study, the cytotoxicity was associated with the high release of Zn^2+^. Among them, 100% extract of Zn/Fe2045 showed slight cytotoxicity, whereas Zn/Fe1045 and Zn/Fe1060 exhibited good cytocompatibility (Figure 2).

To date, no systemic or organ-level toxicity has been reported for biodegradable Fe-based biomaterials [5, 25]. Nevertheless, the local biocompatibility at the biomaterial/cell interface remains a critical consideration. One potential issue for Fe-based alloys as cardiovascular stents is the Fenton reaction occurring during corrosion, as highlighted in several studies [13, 26, 27]. In the presence of dissolved oxygen, iron generates Fe^2+^ and H_2_O_2_, which participate in the Fenton reaction at the surface of materials to generate hydroxyl radicals (·OH) (reactions 4–5) [13]. ·OH is the most powerful oxidant in biological systems, capable of inducing lipid peroxidation, DNA damage and oxidative stress [26].


(4) Fe2++ O2·-+2H+ → Fe3++H2O2

(5) Fe2++H2O2 → Fe3++·OH+OH-

Excessively rapid degradation also leads to excessive metal ion release and increased ROS, resulting in the risk of cytotoxicity. Quantitative analysis of ROS confirmed that faster degradation rates resulted in increased ROS generation (Figure 2B). However, no negative effect on cell viability was observed *in vitro*, possibly due to the short half-life and limited stability of ROS in the extracts.

Compared with Fe, Zn/Fe1060 showed a faster degradation rate during 56 days of implantation, while it is still suboptimal for ideal vascular scaffold applications that require gradual resorption after vascular remodeling (target timeframe: 3–6 months). Notably, cross-sectional SEM demonstrated uniform degradation morphology in the Zn/Fe system (Figure 6). This feature satisfies the basic criteria for bioresorbable flow dividers (BRFD) applications, where materials are expected to maintain mechanical integrity through a uniform and controlled degradation process during endothelialization [28]. Further investigation on the complete degradation kinetics and comprehensive biosafety evaluations is necessary.

### Antioxidant effects of Zn in iron-rich microenvironments

Zn is similar to Fe in biology and is relatively abundant in cells [29]. Numerous studies have established that Zn has antioxidant properties in the organism [19, 29–31]. Zn^2+^ induces metallothionein (MT) synthesis by activating the expression of MT and zinc transporter protein (ZnT) genes through the transcription factor (MTF-1). MT is a redox-active zinc protein that is biochemically coupled to the cellular redox state [32]. It can bind a part of free Fe^2+^ by the thiol group (-SH), thereby preventing Fe^2+^ from participating in the Fenton reaction. Moreover, MT can serve as an efficient antioxidant that directly scavenges free radicals [33]. To validate the antioxidant mechanism, ECs were pretreated with non-toxic Zn^2+^ and then exposed to high concentrations of Fe^2+^ to simulate the corrosion microenvironment. As demonstrated in Figure 3D, pretreatment with 50 μM Zn^2+^ markedly enhanced cell viability compared with cells directly exposed to Fe^2+^ alone. This indicates that Zn supplementation effectively suppressed Fe^2+^-induced oxidative damage in ECs by inducing MT.

**Figure 9:**
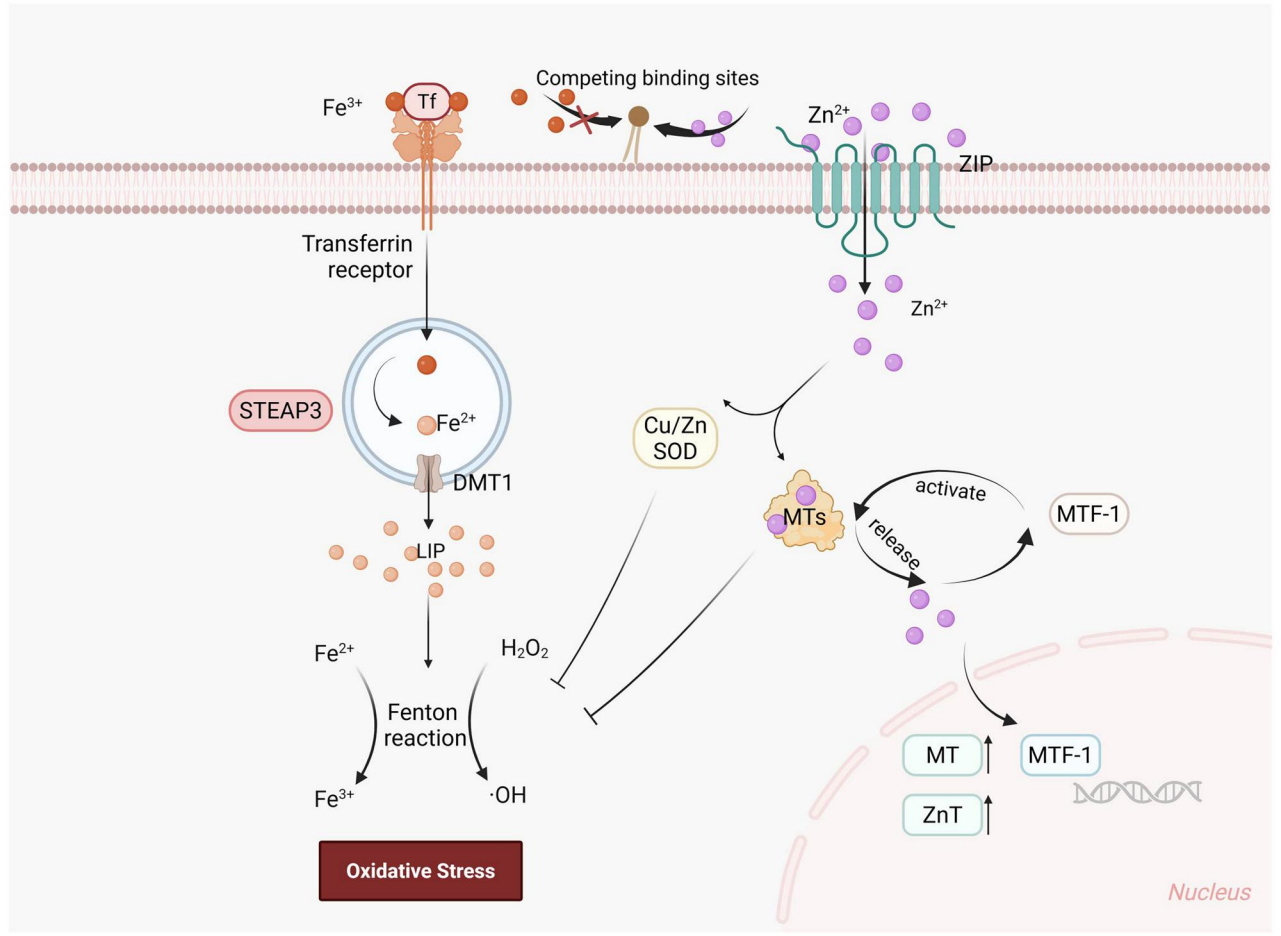
Schematic diagram of the multiple antioxidant mechanisms of Zn/Fe system, created in BioRender.

Yang et al. [20] demonstrated that additional supplementation with 0.02–0.04 mmol/l Zn^2+^ alleviated the accumulation of ROS in HUVECs co-cultured with Fe. Similarly, ECs cultured in Zn/Fe1045 extract exhibited superior viability compared to those cultured in Fe extract (Figure 2C), further supporting the protective role of Zn^2+^ in alleviating Fe-related oxidative stress. Furthermore, Zn^2+^ has been demonstrated to participate synergistically in cellular antioxidant defense through multiple mechanisms (Figure 9). Zn is an important component of the physiological antioxidant membrane network that can competitively occupy key phospholipid-binding sites on the membrane surface of Fe to reduce the intracellular accumulation of Fe^2+^ and prevent the initiation and propagation of lipid oxidation [19, 34]. Zn is also an active component of and promotes the activity of superoxide dismutase (SOD), which directly catalyzes the disproportionation of superoxide anion radicals ($O_{2}^{\cdot -}$) to H_2_O_2_ and O_2_.

### *In vivo* biocompatibility

The lumen area of stented vessels tends to decrease over time due to neointimal hyperplasia. For instance, a 40% reduction in lumen diameter was reported in magnesium-based BRS from 10–35 days of implantation, and clinical trials have confirmed that neointimal growth is one of the main mechanisms of restenosis after stent placement [35, 36]. Similarly, Gao et al. [1] reported narrowing of the mean lumen area in iron-based stents at 9 months, despite observed late lumen enlargement. Ideal bioresorbable stents elute degradation products that reduce SMCs migration and restenosis, while allowing for rapid endothelialization.

To assess the biological response to Zn/Fe implants, a rat common carotid artery (CCA) implantation model was employed. No evidence of thrombosis or acute inflammatory cell infiltration was observed during the initial implantation period. CD-68-labeled macrophages were present around the wires after 10 months, indicating a mild inflammatory response. It is reported that the insoluble iron corrosion products can be phagocytosed by macrophages at the implantation site and transferred to hemosiderin in adventitia, ultimately transferred to the spleen by lymphatic and circulatory pathways, thereby completing the clearance of corrosion products [37].

Notably, a thinner neointima was observed in Zn/Fe1060 compared to Fe after 56 days of implantation (Figure 7). This result is supported by previous studies in which Bowen and Goldman et al. showed that no neointimal thickening was observed after implantation of pure Zn wires into the abdominal aorta of mice; whereas, in the same animal model, pure Fe wires experienced extensive neointimal proliferation [38, 39]. Another study showed that neointimal proliferation was effectively inhibited by exogenous Zn^2+^ treatment in a rat carotid artery injury model [40]. The beneficial antiproliferative effects of Zn^2+^ are primarily attributed to several known biological properties, including (i) reduction of pathological responses after vascular injury by maintaining endothelial cell integrity [41, 42]; (ii) antioxidant and anti-inflammatory effects that suppress ROS-mediated cellular damage [31, 43]; and (iii) inhibition of SMCs proliferation through activation of the caspase-3-dependent apoptotic pathway [44]. Thus, the improved histomorphological performance can be attributed to the Zn-implanted layer, which acts as a slow-release platform for Zn^2+^. This inhibits ·OH oxidative damage to the local tissue, improves the inflammatory microenvironment and inhibits the growth of the neointima. Further long-term studies are required to confirm its sustained efficacy.

## Conclusion

In this study, the *in vitro/vivo* biological responses of Zn ion-implanted absorbable Fe (Zn/Fe) were systematically investigated. *In vitro* cell experiments verified that Zn/Fe exerts an antioxidant effect by releasing Zn^2+^ during the early degradation, thereby improving endothelial cell adhesion and proliferation. During implantation in the rat common carotid artery, Zn/Fe showed accelerated and uniform degradation behavior. After 28 days of implantation, Zn/Fe exhibited a mild inflammatory response and rapid endothelialization. After 56 days of implantation, Zn/Fe significantly mitigated neointimal hyperplasia with a 36.1% reduction in lumen loss compared to pure Fe. These results suggest that the Zn ion-implanted Fe has translational potential in vascular interventional medical devices.

## Supplementary Material

rbaf112_Supplementary_Data
