# Supplementary figures and images for: Zn ion-implanted absorbable Fe for improved cytocompatibility and mitigated neointimal hyperplasia
Source: Regen Biomater. 2025 Nov 7;13:rbaf112. doi: 10.1093/rb/rbaf112 (PMC13020250; doi:10.1093/rb/rbaf112)

**Supplementary materials**


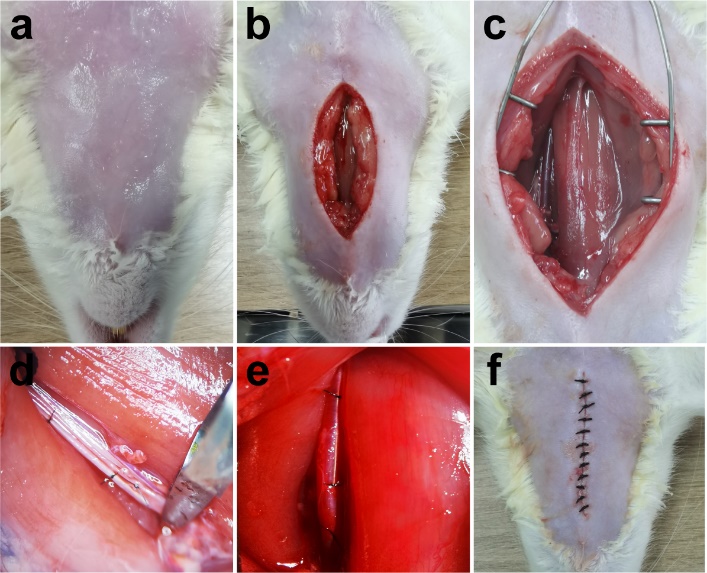


**Figure S1 Surgical procedure.**

Supplement: rbaf112_Supplementary_Data [file rbaf112_supplementary_data.zip › Supplementary material.docx]
